# To Be or Not to Be Mesophilic, That Is the Question for *Aeromonas salmonicida*

**DOI:** 10.3390/microorganisms10020240

**Published:** 2022-01-22

**Authors:** Antony T. Vincent, Steve J. Charette

**Affiliations:** 1 Département des Sciences Animales, Faculté des Sciences de L’agriculture et de L’alimentation, Université Laval, 2425, rue de l’Agriculture, Quebec City, QC G1V 0A6, Canada; antony.vincent@fsaa.ulaval.ca; 2 Département de Biochimie, de Microbiologie et de Bio-Informatique, Faculté des Sciences et de Génie, Université Laval, 1045, av. de la Médecine, Quebec City, QC G1V 0A6, Canada; 3 Institut de Biologie Intégrative et des Systèmes, Pavillon Charles-Eugène-Marchand, Université Laval, 1030, av. de la Médecine, Quebec City, QC G1V 0A6, Canada; 4 Centre de Recherche de l’Institut Universitaire de Cardiologie et de Pneumologie de Québec, Hôpital Laval; Quebec City, QC G1V 4G5, Canada

**Keywords:** *Aeromonas salmonicida*, lifestyle, mesophile, psychrophile, phylogeny, insertion sequence, *vapA*, A-layer, speciation

## Abstract

The bacterium *Aeromonas salmonicida* has long been known to be one of the most feared pathogens in fish farming. However, the more we discover about this bacterial species, the more we question whether it is really exclusively an aquatic pathogen. In recent years, it has become obvious that this bacterial species includes a myriad of strains with various lifestyle and ecological niches, including the well-known strict psychrophiles, the first bacteria known of the species, and the newly described mesophilic strains. The mesophiles are able to grow at low temperatures, but even better at temperatures of approximately 37 °C, which strict psychrophiles cannot do. In this perspective article, we address some aspects surrounding this dual lifestyle in *A.* *salmonicida*, including the impact of mobile genetic elements, and how future research around this bacterial species may focus on the psychrophilic/mesophilic dichotomy, which makes *A.* *salmonicida* an increasingly interesting and relevant model for the study of speciation.

## 1. *Aeromonas salmonicida* Subspecies: A Complex Taxonomic Dilemma

The bacterium *Aeromonas salmonicida* is a well-known pathogen in aquaculture [1]. It first appeared in scientific literature in a German journal in 1890; it was identified as a pathogen of fish and named *Bacillus der Forellenseuche*, which means “trout disease bacillus” [2]. As its name suggests, this bacterium was first thought to be a salmonid-specific pathogen causing furunculosis in these fish. However, subsequently, the range of aquatic hosts infected by bacteria of this species increased significantly with different types of fish being infected such as goldfish, carp, and perch, and this, almost everywhere on the planet and particularly in the context of fish farming [3]. Consequently, it is a pathogen of great importance throughout the world. For example, in Quebec (Canada), this bacterial species is responsible for 25 to 60% of all infections reported in farmed fish [4].

The taxonomy of *A. salmonicida* has been reworked many times since, and now uses a more systematic approach [3]. The broad lines of the taxonomy of *A. salmonicida* have stabilized since 1986, when the *Aeromonadaceae* family was created [5]. Before 2000, the *salmonicida* species was divided into four subspecies: *salmonicida*, *smithia*, *achromogenes* and *masoucida*. All bacteria included in these subspecies were known to be pathogens for various cold-water fish species, with the subspecies *salmonicida* being the most studied and causing furunculosis in salmonids [3]. The subspecies *salmonicida* is consider as typical subspecies while the others are named atypical and are responsible of fish infection outside salmonids [3]. All of these bacteria were considered to be strictly psychrophilic, and therefore unable to grow at 37 °C, although a few exceptions were mentioned in the literature [6,7,8,9,10]. It was therefore reasonable to believe that *A. salmonicida* bacteria were adapted to cold water fish.

The publication of the subspecies *pectinolytica* in 2000 [11] complicated the logic that the bacteria of the species *salmonicida* would be adapted to cold water fish. Bacteria belonging to subspecies *pectinolytica* that were isolated from a polluted river in Argentina, have no known host and can grow efficiently at 37 °C. This unexpected characteristic, however, remained anecdotal in the species *salmonicida*, while psychrophilic bacteria were much more diagnosed and studied [3,9]. The publication of this new subspecies also coincided with a moment of great scientific effervescence: the publication of the human genome [12,13]. However, the sequencing of a genome, even a bacterial one, remained complex and expensive. It was not until 2015 that the DNA of the type strain (34mel^T^) of *A. salmonicida* subsp. *pectinolytica* was sequenced by a high-throughput sequencing method [14]. The publication of this genome validated that subspecies *pectinolytica* belongs to species *salmonicida*.

In 2016, one year after the publication of the genome of the type strain of *A. salmonicida* subsp. *pectinolytica*, the genomes of three other mesophilic strains of *A. salmonicida* were published [15]. Similar to strain 34mel^T^ of the subspecies *pectinolytica*, all three strains originated from a warm region (India), and had no known host, as they were isolated from a public market in Mumbai [15,16]. Since that time, the genomic sequences of several mesophilic strains of *A. salmonicida* have been published and investigated. While the early mesophilic strains had no known host, recently described strains have been isolated from a leech [17], human patients [18], and diseased fish [19] and birds [20].

**Figure 1:**
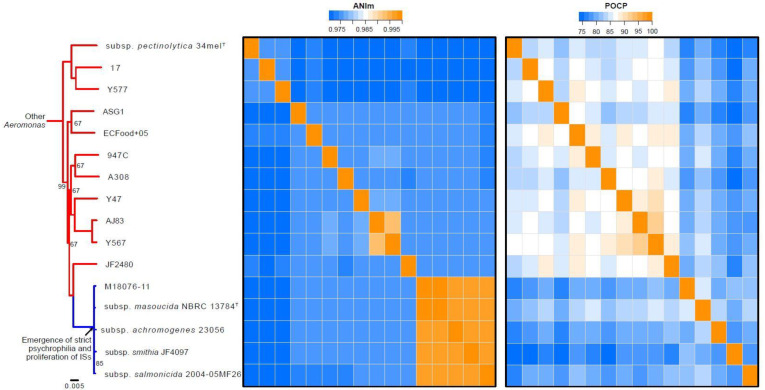
Molecular phylogeny, based on softcore genes, and matrices of ANI and POCP values. The analyses were performed as previously described [20]. Only bootstrap values below 100 are shown. Mesophilic strains are indicated in red and strict psychrophilic strains in blue. The subspecies are indicated when known, otherwise only the names of the strains are reported. All genomic sequences are publicly available on GenBank and the BioSample numbers are: *A. salmonicida* subsp. *pectinolytica* 34mel^T^ (SAMN04208118), *A. salmonicida* 17 (SAMN09062889), *A. salmonicida* Y577 (SAMN03395037), *A. salmonicida* ASG1 (SAMN06461346), *A. salmonicida* ECFood + 05 (SAMN07450651), *A. salmonicida* 947C (SAMN08524351), *A. salmonicida* A308 (SAMN08524352), *A. salmonicida* Y47 (SAMN03395034), *A. salmonicida* AJ83 (SAMN08524353), *A. salmonicida* Y567 (SAMN03395036), *A. salmonicida* JF2480 (SAMN12399690), *A. salmonicida* M18076-11 (SAMN07491057), *A. salmonicida* subsp. *masoucida* NBRC 13784^T^ (SAMD00000014), *A. salmonicida* subsp. *achromogenes* 23,056 (SAMN11836204), *A. salmonicida* subsp. *smithia* JF4097 (SAMN03396265), and *A. salmonicida* subsp. *salmonicida* 2004-05MF26 (SAMN03120845). Other species of *Aeromonas* have been added to root the tree: *A. bestiarum* CECT 4227^T^ (SAMEA2752425), *A. dhakensis* CECT 7289^T^ (SAMEA2752427), *A. veronii* CECT 4257^T^ (SAMEA2752404), *A. rivuli* DSM 22539^T^ (SAMEA2752413), *A. schubertii* CECT 4240^T^ (SAMEA2752410), *A. tecta* CECT 7082^T^ (SAMEA2752406), *A. caviae* CECT 838^T^ (SAMEA2752423), and *A. media* CECT 4232^T^ (SAMEA2752416).

In addition to having a great diversity of hosts, the mesophilic strains of *A. salmonicida* are more genetically diverse than the psychrophilic strains. At the phylogenetic level, this characteristic results in longer branches for mesophilic than psychrophilic strains [18] (Figure 1). This result correlates with the Average Nucleotide Identity (ANI) values, which demonstrates significant genetic proximity in psychrophiles and greater diversity in mesophiles. An analysis of the percentage of conserved proteins (POCP) has shown that the mesophilic strains do, however, have a common gene repertoire, while the different psychrophilic subspecies are more divergent from one another [20] (Figure 1). These results make the taxonomic classification of mesophilic strains difficult. If we follow nucleotide logic, where the different mesophilic strains show greater diversity individually than the psychrophilic subspecies among themselves, this would mean that there would be almost as many mesophilic subspecies as there are strains currently isolated. On the contrary, considering the gene repertoire, there would probably only be one mesophilic subspecies. One of the challenges to fit mesophilic strains in the general taxonomy of *A. salmonicida* comes from the fact that there are relatively well-established criteria to define the different bacterial species (e.g., ANI ≥ 96% and isDDH of ≥70%) [21] while the criteria for the allocation of subspecies remain unclear.

*A. salmonicida* seems to demonstrate that evolution is a long continuum that is difficult to categorize into taxonomic ranks, and that certain bacteria may not meet the criteria currently used. Another example is with *Mycobacterium*, whose species are among the most studied, and yet whose taxonomy is still subject to debate [22,23]. In the case of *A. salmonicida*, it is, however, obvious that it will be necessary to isolate and characterize new mesophilic strains to develop a more representative idea of the real diversity of these strains.

## 2. A Potential Hidden Diversity

The diversity and importance of mesophilic strains are likely underestimated. Mesophilic strains share more characteristics with other species of *Aeromonas* and more particularly with *Aeromonas hydrophila,* such as the absence of pigmentation, growth at 37 °C, and even the architecture of their lipopolysaccharides (LPS) [24,25], than with psychrophilic strains. An example of this is the misidentification of a strain initially identified as *A. hydrophila*, but which turned out to be a mesophilic strain of *A. salmonicida* after analysis of the sequence of its 16S rRNA gene. The initial identification of this strain dates back to decades ago, when molecular methods were not available as they are now [26]. Thus, it is highly probable that many mesophilic *A. salmonicida* strains lurk under other identities in the literature and in laboratory freezers. In the future, therefore, we will likely find new mesophilic strains by exploring different environments, but also by re-exploring already characterized strains.

Based on current data, mesophilic strains seem less problematic for the health of humans or animals than psychrophilic strains, which particularly impact aquaculture. This has led the scientific community to take a preferential interest in these psychrophilic strains, especially since many mesophilic strains of *A. salmonicida* have potentially gone under the radar as mentioned above. So, are we not seeing the wood for the trees? Has the biased study of psychrophilic strains prevented a full awareness of the extent and diversity of mesophilic strains?

There is still a lot of work to do to get a true picture of the situation. It also implies that we must remain open to the idea of finding mesophilic *A. salmonicida* strains in unexpected environments distinct from those where psychrophilic strains are found. Mesophilic strains may also be hidden in environments where psychrophilic strains are usually found; an example is strain SRW-OG1 isolated from fish [19]. To support the idea that mesophilic strains can inhabit cold environments, it is important to remember that these strains have been shown to have a higher growth capacity than typical psychrophilic strains at 18 °C and even 7 °C [15].

Considering that mesophilic strains have better growth than psychrophilic strains, even at low temperature, the abundance of psychrophilic strains can potentially be explained by the principle of r/K selection [27]. Indeed, mesophilic strains would be r-strategists that favor growth without particular specialization, while psychrophilic strains, under evolutionary pressure, would have become K-strategists well adapted to specific aquatic hosts displaying limiting growth conditions not favorable to mesophilic strains.

Since mesophiles can grow at both high and low temperatures, while psychrophiles grow only at low temperatures, it would be more precise to name this last group “strict psychrophiles” to help recognize the much wider growth temperature range of mesophiles. We will use the “strict” adjective for the rest of this article.

## 3. Insertion Sequences: Selfish Elements Are Not So Selfish after All

Among all the *Aeromonas* species, only the *salmonicida* species presents psychrophilic strains so far [24]. If we consider the point mentioned above, that the mesophilic strains are more efficient than the psychrophilic strains at low temperature, one might wonder if, in fact, the strict psychrophilic strains are not variants of the original mesophilic strains, rather than being the archetype of the species *salmonicida*. The phylogeny also suggests this, with a late appearance of strict psychrophilic strains compared to mesophilic strains (Figure 1). What caused this shift from one lifestyle type to another? The strict psychrophilic strains appear more likely to be strains that have lost some ability (i.e., growth at high temperature). What might have caused this evolution?

Approximately 40 years ago, researchers demonstrated that culturing bacteria of the subspecies *salmonicida* at high temperature causes a loss of virulence, which was linked to the loss of the A-layer on the surface of the cells [28]. The loss of this protein layer was explained in some cases by the transposition of insertion sequences (ISs) in the *vapA* gene, which codes for the protein that forms the A-layer; and also in the adjacent *abcA* gene which codes for a protein involved in *O*-polysaccharide synthesis and transport, and consequently for the biogenesis of the A-layer attached to LPS [29,30,31]. ISs are mobile genetic elements that have the ability to move from one target sequence to another or that can expand within the genome that becomes “infected” by these parasitic genetic elements [32]. The genome of psychrophilic strains is particularly rich in ISs [15,33]. We can find up to hundred copies of various classes of ISs inside a chromosome which is approximately 4.7 Mb, but also in plasmids, which are autoreplicative mobile genetic elements particularly common in strict psychrophilic strains [33,34,35].

ISs contribute to great genetic instability in strict psychrophilic strains. Certain ISs in plasmid pAsa5, found in all *salmonicida* subspecies strains, serve as a substrate for a still-unknown mechanism that causes the rearrangement of the plasmid when the bacterium is exposed to temperatures of 22 °C and above [36,37,38,39,40]. The plasmid pAsa5 carries, among other things, the majority of genes of the type three secretion system (TTSS). This system is essential for the virulence of the bacteria in fish [41]. The rearrangement of the plasmid causes the loss of the TTSS locus, and the loss of the virulence of the bacteria [36,37]. Thus, ISs would have caused, at least in part, the loss of the mesophilic trait of the bacteria by disrupting key genes. ISs would also now be a lock that prevents strict psychrophilic strains from becoming virulent mesophilic strains again, among other things, by the loss of TTSS when bacteria are grown at temperatures of 22 °C and above.

**Figure 2:**
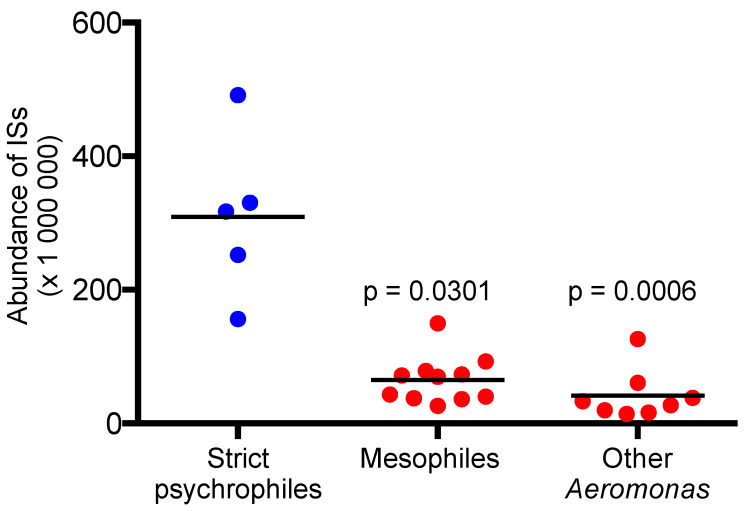
Percentage of sequencing reads mapped by diamond (blastx algorithm) against the database of insertion sequence transposases provided by Prokka [45]. The values were standardized to consider the lengths of the proteins and the depth of the datasets, as was done previously [15]. The same dataset was used as for the phylogenetic analysis (Figure 1). A Kruskal–Wallis test with a Dunn’s multiple test comparison was performed using PRISM 6 (GraphPad).

IS expansion is a trait common to bacteria that establish a dependency relationship with their host, which is likely the case for *A. salmonicida* psychrophilic strains, considering the abundance of ISs in their genome [32]. Mesophilic strains have been reported to have fewer ISs than strict psychrophilic strains of *A. salmonicida* [15,42]. Since these studies, many sequences from other mesophiles have been published [17,18,20]. By re-evaluating the quantity of ISs, as previously described elsewhere [15], it is possible to observe that mesophilic strains definitely have fewer ISs than psychrophilic ones (Figure 2).

Moreover, by analyzing other species of *Aeromonas* representative of the diversity of the genus according to the study of Colston et al., we note that relatively few ISs are found in these genomes, similar to mesophilic strains of the species *salmonicida* [21] (Figure 2). This result suggests that the expansion of ISs co-occurs with strict psychrophilia in *A. salmonicida*.

Although studies have made it possible to demonstrate that the few ISs found in mesophilic strains are generally not the same as in the genomes of psychrophilic strains [15,42], several other completely assembled genomes will be required in the future to more precisely establish the repertoire of ISs in mesophilic strains. This is particularly true in the context where it has been shown that ISs, by virtue of being repeated, are among the main reasons for assembly breakdown in *A. salmonicida* [40,43,44]. The increasing accessibility to long-read sequencing technologies (PacBio and Oxford Nanopore) will accelerate research on the role of ISs in the evolution of *A. salmonicida*. These technologies will also shed light on other mobile DNA elements, such as plasmids, which for the moment have been very little described and characterized in mesophilic strains [35].

## 4. Other Villains to Consider?

The presence of ISs alone is probably not enough to explain the transition of mesophiles to the strict psychrophilic restrained lifestyle. One can imagine that genes could also have been acquired by bacteria, pushing them towards the loss of the mesophilic phenotype with, in exchange, a gain in their virulence in a very specific context. The study of Indian strains in 2016 addressed this aspect and suggested a greater number of genes under evolutionary pressure in mesophilic strains compared to psychrophilic strains [15]. This would contradict the last statement. However, some genes could have a greater impact on their own than others, by being responsible for phenotypic traits or cell structures that limit the temperature at which the bacteria can grow.

Among these structures, the A-layer seems to be an element worth some attention. The *vapA* gene that codes for the A-layer protein is only found in strict psychrophilic strains [46,47]. The A-layer is a protein structure that plays a role in the virulence of the bacterium [28]. Due to the A-layer’s important relative proportion in the proteome of the bacterium, it could cause a significant metabolic burden that limits the growth of bacteria [48]. The A-layer could, by interacting with the LPS and therefore the membrane of the bacteria, also have an impact on the fluidity of the membrane and limit its integrity or its functionality at higher temperatures [29]. The fact that A-layer is lost in strict psychrophilic bacteria grown at higher temperatures is an argument in favor of this hypothesis [28,31]. Experimental work is needed to confirm this hypothesis of the involvement of A-layer in limiting the growth temperature of strict psychrophilic strains.

Another element points to a link between the lifestyle of strains and the composition of their cell wall: bacteriophages [26,49]. To date, phages that infect species *Aeromonas salmonicida* have demonstrated a host spectrum that appears to be dictated primarily by the lifestyle of the bacteria. Typically, phages infect strict psychrophilic strains but not mesophilic strains, and vice versa, with the exception of phage T7-Ah which can infect mesophilic strains and a specific subgroup of psychrophilic strains [26,50]. However, before drawing definitive conclusions about lifestyle-influenced interactions with viruses, it will be necessary to characterize in detail a greater number of phages that target *A. salmonicida* and their interactions with both types of bacteria.

The existence of mesophilic and strict psychrophilic strains raises many questions:

1. Can strict psychrophilic strains be manipulated to become mesophilic again?
2. Would a simple gradual growth at higher and higher temperatures allow a permanent return to a mesophilic lifestyle for these strains through the appearance of mutations or other more significant genetic alterations?
3. Is it possible that epigenetics can also play a complementary role in this phenomenon by providing a temporary adaptation to temperature variations?
4. What was the starting point for the distinction between mesophilic and psychrophilic strains?

For this last question, we wish to attempt an answer while being aware that it is speculative. As stated earlier, mesophilic strains characterized so far have few or no plasmids, while strict psychrophilic strains typically have several [35]. It is conceivable that genetic elements specific to strict psychrophilic strains were acquired via plasmids that initially carried these genetic elements (ISs, *vapA* gene, etc.). This scenario is particularly plausible for ISs that would have used the plasmids as a gateway to invade the genome of the ancestral bacterium that gave the current strict psychrophilic strains, and then over time, caused various modifications that led to a more limited lifestyle [32].

The study of the species of *A. salmonicida* goes beyond the simple aspect of its pathogenesis, its resistance to antibiotics, or the treatments that may be designed to fight the infections it causes. This bacterial species represents a model of choice for studying the mechanisms and consequences of speciation. One benefit of future research on *A. salmonicida* is that it will surely make it possible to learn more about this subject while confirming the great diversity of strains of this bacterial species.

## Data Availability

Not applicable.
